# Proteomics informed by transcriptomics for characterising differential cellular susceptibility to Nelson Bay orthoreovirus infection

**DOI:** 10.1186/s12864-017-3994-x

**Published:** 2017-08-14

**Authors:** Lawrence Mok, James W. Wynne, Mary Tachedjian, Brian Shiell, Kris Ford, David A. Matthews, Antony Bacic, Wojtek P. Michalski

**Affiliations:** 1 CSIRO, Australian Animal Health Laboratory, East Geelong, VIC Australia; 2 ARC Centre of Excellence in Plant Cell Walls School of BioSciences, The University of Melbourne, Melbourne, VIC Australia; 3 Department of Cellular and Molecular Medicine, School of Medical Sciences, University of Bristol, Bristol, UK

**Keywords:** Proteomics, Transcriptomics, Proteomics informed by transcriptomics, PIT, Immune, Host, Response, Nelson bay, Orthoreovirus, Interferon

## Abstract

**Background:**

Nelson Bay orthoreovirus (NBV) is a fusogenic bat borne virus with an unknown zoonotic potential. Previous studies have shown that NBV can infect and replicate in a wide variety of cell types derived from their natural host (bat), as well as from human, mouse and monkey. Within permissive cells, NBV induced significant cytopathic effects characterised by cell-cell fusion and syncytia formation. To understand the molecular events that underpin NBV infection we examined the host transcriptome and proteome response of two cell types, derived from bat (PaKiT03) and mouse (L929), to characterise differential cellular susceptibility to NBV.

**Results:**

Despite significant differences in NBV replication and cytopathic effects in the L929 and PaKiT03 cells, the host response was remarkably similar in these cells. At both the transcriptome and proteome level, the host response was dominated by IFN production and signalling pathways. The majority of proteins up-regulated in L929 and PaKiT03 cells were also up-regulated at the mRNA (gene) level, and included many important IFN stimulated genes. Further functional experimentation demonstrated that stimulating IFN signalling prior to infection, significantly reduced NBV replication in PaKiT03 cells. Moreover, inhibiting IFN signalling (through specific siRNAs) increased NBV replication in L929 cells. In line with the significant cytopathic effects seen in PaKiT03 cells, we also observed a down-regulation of genes involved in cell-cell junctions, which may be related to the fusogenic effects of NBV.

**Conclusions:**

This study provides new multi-dimensional insights into the host response of mammalian cells to NBV infection. We show that IFN activity is capable of reducing NBV replication, although it is unlikely that this is solely responsible for the reduced replication of NBV in L929 cells. The molecular events that underpin the fusogenic cytopathic effects described here will prove valuable for identifying potential therapeutic targets against fusogenic orthoreovirus.

**Electronic supplementary material:**

The online version of this article (doi:10.1186/s12864-017-3994-x) contains supplementary material, which is available to authorized users.

## Background

Nelson Bay orthoreovirus (NBV) is the prototypic member of the *Pteropine orthoreovirus* species. This group contains viruses that have been isolated from both bats and humans. NBV was isolated in 1970 from the blood of a grey-headed flying fox (*Pteropus poliocephalus*) [1]. Bats are considered natural hosts for a number of *Pteropine orthoreovirus* species, including NBV, Pulau virus [2] and Xi River virus [3]. Human isolates of *Pteropine orthoreovirus* species are genetically related to NBV [4] and are often from patients with respiratory illness. Indeed, Melaka virus (MelV) was the first bat-related orthoreovirus isolated from a human exhibiting respiratory symptoms [5] with the transmission believed to be from bats. Further, other *Pteropine orthoreovirus* species such as Kampar, HK23629/07, HK46886/09, HK50842/10 and Miyazaki-Bali/2007 have all been isolated from humans presenting respiratory illness [6–9]. A link to either direct or indirect contact with bats was demonstrated in a number of these cases [5, 6, 8, 10]. A seroprevalence study of 272 human serum samples in Vietnam identified 12 serum samples to be seropositive for *Pteropine orthoreovirus*. This suggests that human infection with *Pteropine orthoreovirus* species is potentially more prevalent than initially thought [11]. To date, NBV has not been linked to clinical disease in humans, however given the increasing number of spill-over events from bats to humans, the potential transmission – and pathogenicity – of this virus in humans, domestic animals and livestock must be considered.

Previous work within our laboratory has shown that NBV generally displays a broad species tropism, and is capable of infecting various mammalian cell types derived from diverse mammalian taxa, including human, mouse, monkey (Vero) and its presumed reservoir host, the Australian black flying fox (*Pteropus alecto*) [12]. Within almost all of these cell lines, NBV - like other fusogenic orthoreoviruses – causes extensive cell-cell fusion (syncytia) and viral replication. One exception was a mouse fibroblast cell line, known as L929, which was significantly less permissive to infection compared to other mammalian cell lines [12]. In NBV infected L929 cells, there was reduced syncytia and a difference in viral replication compared to permissive cell types, including other mouse cell types. The ability of L929 cells to resist infection with NBV was an interesting observation, given that L929 is also the cell type of choice that is used to propagate the non-fusogenic mammalian orthoreovirus (MRV) [13].

The L929 cells are also highly permissive to other fusogenic orthoreoviruses such as Miyazaki-Bali/2007 (MB) [9], Pulau and Melaka [12]. In particular, MB induces large syncytia in L929 and Vero cells as early as 12 h post infection (hpi) [9], and MB replicates to similar titres in L929 and Vero cells. Taken together, these findings suggest that the reduced permissiveness of L929 cells to NBV is due to a unique host/pathogen interaction between the L929 cell type and NBV. Presently the molecular mechanisms that underpin this interaction is unknown. We propose that elucidating the host factors that regulate the interaction between L929 cells and NBV will shed light on the elements required for an effective antiviral response against fusogenic orthoreoviruses, thereby informing future therapeutic design.

Fusogenic orthoreoviruses induce cell-cell fusion through their fusion protein, which is also known as fusion associated small transmembrane (FAST) proteins [14]. These are the smallest known viral fusion proteins [15] with molecular weights ranging from 10 to 15 kDa [16, 17]. Unlike other fusion proteins these are not involved in virus entry or exit. The induction of syncytia is not required for virus entry and is only observed during infection with the FAST protein trafficked to the plasma membrane, mediating fusion with neighbouring cells [18]. These fusion proteins provide an efficient way for dissemination of the virus via cell to cell spread. The fusion of uninfected cells, allow for the use of their translational machinery for virus replication. In addition, syncytia formation serves to sequester the virus allowing it to avoid immune system components that attempt to clear infection, such as complement proteins, antibodies and phagocytes [19, 20].

The generation of monoreassortant fusogenic othoreoviruses, obtained through reverse genetics, have been used to define the role of specific viral proteins, including the fusion protein, in L929 cells. Indeed, no difference in replication kinetics was observed when the S1 segment of MB was replaced with the S1 segment of NBV [9]. The recombinant monoreassortant MB virus demonstrated similar replication kinetics compared to the wild-type MB in L929 cells, thereby indicating that the NBV S1 segment, which includes the p10 fusion protein along with the attachment protein σC - is functionally maintained in L929 cells. This led us to hypothesise that host factors rather than viral proteins, are likely to play a role in determining the permissiveness of L929 cells to NBV.

**Fig. 1:**
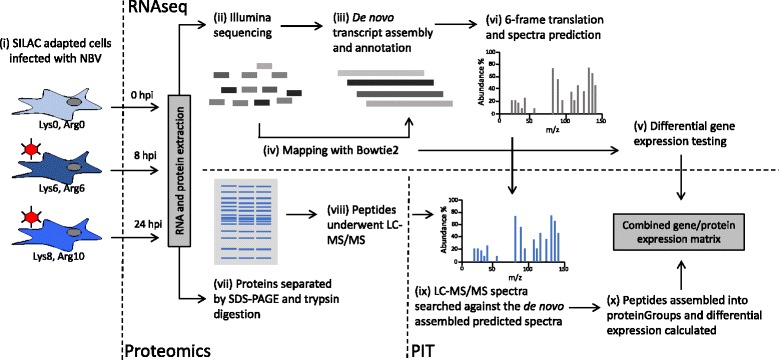
Proteomics Informed by Transcriptomics workflow. (i) Cells are adapted to SILAC media containing Lysine and Arginine with isotopes of Carbon and Nitrogen. Cells were infected with NBV for 0 (control), 8 and 24 h using an MOI of 1.5. (ii) Total RNA was isolated and mRNA sequenced, using 100 base pair paired-end reads. (iii) Reads obtained from sequencing, were quality trimmed and transcripts assembled *de novo* using Trinity. Assembled transcripts were annotated using BLASTx against the non-redundant UniProtKB/SwissProt protein database. (iv) Sequence reads are mapped back to the *de novo* assembled transcriptome using Bowtie2. (v) Differential gene expression testing was calculated with expression values relative to 0 h control determined by DESeq. (vi) The *de novo* transcriptome was translated in 6 frames as a database for MS. (vii) Extracted proteins were separated by SDS-PAGE and digested via in-gel trypsin digestion. (viii) Peptides were analysed by LC-MS/MS and the (ix) MS spectra searched against the 6-frame translated *de novo* transcriptome. (x) Peptides are compiled into proteinGroups and the differential expression is calculated using MaxQuant

The molecular events underlying NBV mediated cell-cell fusion and subsequent viral replication are not fully understood. The mechanisms by which L929 cells limit syncytia formation and viral replication is of particular interest in the broader context of understanding, and ultimately mitigating, infection with fusogenic non-enveloped viruses. To this end, the present study aimed to identify host factors that influence NBV replication and cytopathic effects by exploiting differences in NBV permissiveness between cell types. Immortalised bat kidney cells, PaKiT03, which represent a highly permissive (and natural host) cell type was compared with the relatively resistant L929 cells. To identify critical host factors, we used an integrated Proteomics Informed by Transcriptomics (PIT) approach [21] to simultaneously quantify changes in gene expression and protein synthesis at a genome wide scale. Based on transcriptome sequencing (RNASeq) and stable isotope labelling with amino acids in cell culture (SILAC) coupled with mass spectrometry (MS), this approach has been used to successfully examine virus host interactions previously in a non-model species (Fig. 1) [22]. More recently the PIT approach was evaluated for proteomic characterisation of an organism’s repertoire of genetic transposable elements [23].

Combining different ‘omic methodologies to answer biological questions is necessary to provide different layers of information [24]. For instance, genomics provides information on the number of genes and sequence information of these genes, and using these sequences it is possible to construct phylogenetic trees to compare evolutionary relationships. The application of transcriptome technologies is for the study of an organism’s transcriptome to assess gene expression activities. Although, transcript information is obtained it fails to provide complete information on protein synthesis and their abundance, which is known to not have a perfect correlation to gene expression through transcription. This is particularly true for proteins that have functional (enzymes) other than a structural role in any biological system. Although an integrated PIT approach does not guarantee complete correlation between gene expression and protein synthesis which is due to the temporal lag in the sequence of events. It is considered the most representative approach in comparison with transcriptomics and proteomics applied alone.

## Methods

### Maintenance and SILAC adaption of L929 and PaKiT03 cell types

Immortalised *Pteropus alecto* kidney cells [25] and L929 cells which were not used in SILAC experiments were maintained as follows: PaKiT03 cells were grown in DMEM Nutrient Mixture F-12 Ham with 15 mM HEPES pH 7.4 (Sigma) and L929 cells were grown in MEM with 10 mM HEPES pH 7.4 and 2 mM L-glutamine (Life Technologies) both supplemented with 10% v/v foetal calf serum (FCS).

For SILAC adaption, PaKiT03 and L929 cell types were maintained in Minimal Essential Media (MEM, Thermo Scientific) deficient in L-Lys and L-Arg, supplemented with 10% (v/v) dialysed FCS and differing combinations of L-Lys and L-Arg containing stable isotopes of carbon and nitrogen. L-Lys-2HCl + L-Arg-HCl (unlabelled, designated ‘Light’), ^13^C_6_ L-Lys-2HCl + ^13^C_6_ L-Arg-HCl (‘Medium’) and ^13^C_6_^15^N_2_ L-Lys-2HCl + ^13^C_6_^15^ N_4_ L-Arg-HCl (‘Heavy’). Cells were passaged for five doublings, splitting at 1:10 into T25 (25cm^2^) flasks.

### Verification of correct incorporation of isotope labelled amino acids in cell proteins

Cells from isotope labelled cultures were tested for correct incorporation of isotopic labelled amino acids into proteins. Equal quantity of ‘Light’, ‘Medium’ and ‘Heavy’ cell lysates were analysed by SDS-PAGE in MOPS buffer (Life Technologies) and two randomly selected regions were excised from the gel for in-gel trypsin digestion and MS analysis.

### Infection of adapted cells with NBV

SILAC adapted L929 and PaKiT03 cells were seeded at 5 × 10^6^ cells into T25 flasks (in triplicate) containing SILAC medium with appropriate isotope labelled amino acids and supplemented with 10% (v/v) FCS. Following the overnight incubation at 37 °C the medium was removed and cells were washed with sterile PBS. Both cell cultures were infected with NBV at a MOI of 1.5 for 1 h at 37 °C with gentle rocking every 15 min. The inoculum was removed and cells were washed with PBS. The inoculum was replaced with 4 ml of SILAC isotope labelled media. To confirm infection, SILAC adapted cells were also seeded into glass coverslips in 24 well plates at a density of 30,000 cells/well and infected with NBV as described above. At 0, 8 and 24 hpi cells were fixed in 4% paraformaldehyde and immunofluorescence detecting NBV sigma (σ) proteins were performed with the antiserum for NBV σ2 and σNS proteins as described previously [12]. In each cell line, the viral infection was quantified as the number of nuclei per syncytium, as previously described [12]. The average number of nuclei per syncytium was calculated using a minimum of two replicates and statistical significance tested using a Student’s t-test.

### Sampling of NBV infected L929 and PaKiT03 cells

At the sampling time points of 0, 8 and 24 hpi with NBV, the medium was poured off and cell monolayers washed with PBS. Cells were then trypsinised and harvested after inhibiting trypsin with medium containing 10% v/v FCS. Cells pelleted by centrifugation at 1500 x *g* for 5 min were resuspended in 500 μl of PBS. An aliquot of 100 μl was pelleted by centrifugation as above and lysed in 350 μl of Buffer RLT (Qiagen) for RNA extraction and Real-Time PCR. The pellet of 400 μl aliquot was lysed in SDS-PAGE sample buffer (Life Technologies) for electrophoretic analysis.

### Extraction of RNA for RNA sequencing

All cell lysates in Buffer RLT were homogenised with a QIAShredder and subjected to RNA extraction. The extraction was completed with the Qiagen RNeasy Mini Kit with RNA eluted in 30 μl of nuclease free water (Promega). The concentration of each preparation was determined by NanoDrop (Thermo Scientific) and Qubit RNA quantitation. The RNA integrity was determined following separation on a 0.9% agarose gel 40 mM Tris, 20 mM acetic acid, 1 mM EDTA pH 8.0. An aliquot of 1-3 μg of total RNA (stored on dry ice) was sequenced on Illumina HiSeq 2000 (100 bp paired end reads) at the Australian Genome Research Facility (AGRF, Parkville, Australia).

### Sample preparation for SILAC analysis

For SILAC analysis of infected cells, equal quantities of cell lysates of the ‘Light’, ‘Medium’ and ‘Heavy’ labelled PaKiT03 and L929 were pooled and separated by 4-12% SDS-PAGE in MES buffer (Life Technologies). The samples were stained with Coomassie blue and de-stained overnight. Pooled lanes for PaKiT03 and L929 SILAC-labelled cell lysates were excised into 10 (Replicate 1) and 15 (Replicate 2) equal portions. The proteins within each gel piece were then subjected to in-gel trypsin digestion for MS analysis as described previously [26].

Peptides were analysed by MS on a LTQ Orbitrap Elite (Replicate 1, Thermo Scientific) and LTQ Orbitrap Velos (Replicate 2, Thermo Scientific). Both machines were equipped with a nanoESI interface with an Ultimate 3000 RSLC nano-HPLC (Dionex Ultimate 3000) and tandem Dinoex-C18 columns (Acclaim Pepmap nano-trap (100 Å, 75 μm × 2 cm and Acclaim Pepmap RSLC 100 Å, 75 μm × 15 cm). With the LTQ Orbitrap Elite, the peptides were separated on to the nano-trap column with 0.1% (v/v) formic acid in 3% (v/v) CH_3_CN (5 μl/min for 5 min) before the enrichment column was switched in-line. For elution with 0.1% (v/v) formic acid (solvent A) and 0.l % (v/v) formic acid in 100% (v/v) CH_3_CN (solvent B). The flow gradient was: (i) 0-5 min at 3% B, (ii) 5-6 min, 3-6% B (iii) 6-18 min, 6-10% B (iv) 18-38 min, 10-30% B (v) 38-40 min, 30-45% B (vi) 40-42 min 45-80% B (vii) 42-45 min at 80% B (vii) 45-46 min, 80-3% B and (viii) 46-53 min at 3% B. The LTQ Orbitrap Elite was operated in the data-dependent mode with nanoESI spray voltage of 2.0 kV, capillary temperature of 250 °C and S-lens RF value of 55%. All spectra were acquired in positive mode with full scan MS spectra scanning from m/z 300-1650 in the FT mode at 240,000 resolution after accumulating to a target value of 1.0e^6^. Lock mass of 445.120025 was used. The top 20 most intense precursors were subjected to collision induced dissociation (CID) with normalised collision energy of 30 and activation q of 0.25. Dynamic exclusion of 45 s was applied for repeated precursors. The protocol used for peptide analysis on the LTQ Orbitrap Velos has been described previously [21].

### *De novo* assembly of RNASeq transcriptome and differential gene analysis

RNAseq transcriptome 100 bp paired end sequencing was performed in triplicate (three biological replicates) at 0, 8 and 24 hpi, for both PaKiT03 and L929 cells. FASTQ reads were first groomed using FASTQ Groomer (v. 1.0.4) [27] to evaluate reads quality, reject low quality reads and ensure correct formatting for down-stream processing. The total number of post-filtered reads for each replicate/sample are presented in Additional file 1: Table S1. Transcripts for PaKiT03 and L929 samples were assembled *de novo* using all sequence reads (i.e. 0, 8 and 24 hpi samples) in Trinity [28]. To annotate the assembled transcriptomes, a BLASTx searched against the non-redundant UniProtKB/SwissProt protein database was performed for each transcript. Only transcripts with a BLASTx e-value ≤1.0^−5^ were annotated with gene names. To perform differential gene expression analysis the paired groomed FASTQ read files were mapped to the *de novo* assembled transcriptome with Bowtie2 (v. 0.2) [29]. A summary of mapping statistics is provided as Additional file 1: Table S1. The number of reads mapping to each transcript was quantified using SAMtools [30] for each sample/replicate. Differentially expression testing was performed using the R package DESeq [31]. Only transcripts with an adjusted *p*-value ≤0.05 and a fold change ≥2 up- or down-regulated were considered statistically significant. Sequenced reads from L929 and PaKiT03 samples were also mapped back to the reference NBV genome with Bowtie2 (v. 0.2) [29]. Differential expression was not performed on these samples.

### Quantitative analysis of SILAC mass spectra with MaxQuant

To quantify protein changes following NBV infection in PaKiT03 and L929 cells mass spectra files from the LTQ Orbitrap Velos and LTQ Orbitrap Elite were loaded into MaxQuant (v. 1.4.1.2), with the following group specific parameters: ‘type: standard – 3 labels, ‘Light’ -, ‘Medium’ – Lys 6 and Arg 6, ‘Heavy’ – “Lys 8 and Arg 10 and requantify – ticked”. In global parameters the FASTA files used were all frames translation of the *de novo* assembled transcriptome as previously described [22]. A decoy database entailing a reversed transcriptome was also translated in all frames.

Individual proteinGroup files, containing information on the MaxQuant identified proteins, were obtained from each replicate (*n* = 2) for PaKiT03 and L929 samples. ProteinGroups identified in both replicates (thus two or more peptides) were extracted along with the corresponding abundance ratios at 8 (Medium/Light) and 24 (Heavy/Light) hpi. The proteinGroup lists were then filtered to remove common contaminants (keratin) and those derived from the decoy database. Using BLASTx information for each transcript, the proteinGroups that contained ambiguous protein ID’s were removed. The average ratio was calculated from the two replicates for each protein. Proteins were assessed as differentially regulated if the fold change was ≥2 in any direction. Mass spectra files were also searched against the all frame translation of NBV nucleotide sequences and using the same parameters as above in MaxQuant [4].

### Pathway analysis

Following BLASTx analysis, the PaKiT03 and L929 transcripts were annotated with UniprotKB/Swiss-Prot entry names. The official gene symbols were obtained by converting these entry names to the official gene symbol with the Database to Database conversion from the biological DataBase network [32]. Lists of differentially expressed genes were compiled for each cell line at 8 and 24 hpi. Using the official gene symbol, transcript redundancy was removed for each list (i.e. duplicate transcripts removed). Gene sets were then analysed using the Reactome database [33, 34] to identify overrepresented pathways. For both PaKiT03 and L929 cells the human database was used. The false discovery rate (FDR) is presented for each enriched pathway.

### Real-time quantitative PCR

Purified RNA was converted into First-Strand cDNA using SuperScript II Reverse Transcriptase (Life Technologies) according to manufacturer instructions. The cDNA was then analysed by the SYBR Green Real-Time PCR (Life Technologies). Primer design was completed using Primer3 with the following conditions: “Primer size – 20 bp, Primer melting point - 60°C, Primer G-C content – 55 % and Product size – 100 bp minimum, 150 bp optimum, 200 bp maximum”. The final PCR reaction volume was 20 μl with forward and reverse primer concentrations at 10 μM (Additional file 1: Table S1). The templates used were either mouse, bat or NBV cDNA. The cycle conditions were: “Holding stage - 50°C for 2 min and 95°C for 2 min; Cycling stage - 95°C for 15 sec and 60°C for 1 min for 40 cycles and a melt curve stage of 95° for 15 sec, 60°C for 1 min and 95°C for 15 sec” on an ABSciex StepOne 7500 Plus Real-Time PCR System.

The real-time PCR threshold cycle (Ct) data were exported into an Excel spreadsheet and the Ct value differences between samples were calculated with the relative expression software tool (REST) [35] where the expression levels of target genes were normalised against a reference gene, *Gapdh*.

### Stimulation of PaKiT03 cells with universal type I IFN

PaKiT03 cells were seeded at a density of 3 × 10^4^ cells in 96 well microplates and left overnight at 37 °C to adhere. The cells were stimulated (in triplicate) with 5000 units of Universal Type I IFN (UIFN, PBL Assay Science) in 100 μl of serum free medium, DMEM Nutrient Mixture F-12 Ham with 15 mM HEPES pH 7.4 (Sigma) for 3 h. Control cells were mock stimulated with serum free medium only for 3 h. Following the 3 h stimulations media were removed and both UIFN treated and controls cells were infected NBV at a MOI of 10 for 1 h. The cells in the microplate were washed with sterile PBS and supplied with fresh DMEM Nutrient Mixture F-12 Ham, 15 mM HEPES pH 7.4, with 2% FCS. At 24 and 40 hpi the entire 96 well microplate was frozen at −80 °C for virus titration as previously described [12]. UIFN stimulated and control PaKiT03 cells were also prepared in 24 well microplates for immunofluorescence microscopy as described previously [12]. Earlier time points were not tested as they had previously been shown to be negative for viral proteins. The average number of nuclei per syncytium was calculated using a minimum of two images and differences calculated using the Student’s t-test.

### *Ifnar1* knockdown in L929 cells

The knockdown of the type I IFN receptor, Ifnar1, in L929 cells was performed using siRNAs targeted to *Ifnar1* (siGENOME SMARTpool Mouse Ifnar1, 15,975, Dharmacon)*.* The L929 cells were seeded at a density of 3 × 10^4^ cells per well in a 96 well microplate in duplicate and left overnight at 37 °C to adhere. L929 cells were transfected for 48 h with 60 nmol of siRNA Ifnar1 (Dharmafect, Dharmacon). To quantify the level of mRNA knockdown, following transfection the supernatants were discarded and cells lysed with 200 μl of Buffer RLT. RNA was extracted and gene expression of *Ifnar1* following transfection with siIFNAR1 was assessed with qPCR and differences calculated using the Student’s t-test.

To assess the effect of *Ifnar1* knockdown on NBV replication the L929 cells were seeded and transfected for 48 h as previously described. Following transfection, the supernatants were discarded and the cells infected with NBV at a MOI of 10 with inoculum removed after 1 h. At 24 and 40 hpi the microplates were frozen at −80 °C for virus titration. Virus titration was performed in Vero cells as previously described [12]. In parallel, knockdown (siIFNAR1+) and non-specific control siRNA knockdown L929 cells in 24 well microplates were prepared for immunofluorescence microscopy, for the detection of NBV σ proteins as described previously [12]. Earlier time points were not tested as it has previously been shown that at these time points, viral proteins were not detectable. The average number of nuclei per syncytium was calculated from a minimum of two images using the Student’s t-test.

## Results

### L929 cells are less susceptible to NBV infection

L929 and PaKiT03 cells were adapted to SILAC medium containing amino acids, L-Arg and L-Lys labelled with different isotopes of carbon and nitrogen. The incorporation of ^13^C_6_ L-Lys-2HCl + ^13^C_6_ L-Arg-HCl (‘Medium’) and ^13^C_6_^15^N_2_ L-Lys-2HCl + ^13^C_6_^15^ N_4_ L-Arg-HCl (‘Heavy’) was confirmed by MS before virus infection. Compared to the ‘Light’ cells (^12^C_6_, ^14^N_7_) peptides derived from the ‘Medium’ and ‘Heavy’ adapted cells showed the expected mass-to-charge (m/z) shift of 6 and 8 Da for lysine fragments, respectively, and 6 and 10 Da shift for arginine fragments, respectively (Additional file 2: Figure S1).

**Fig. 2:**
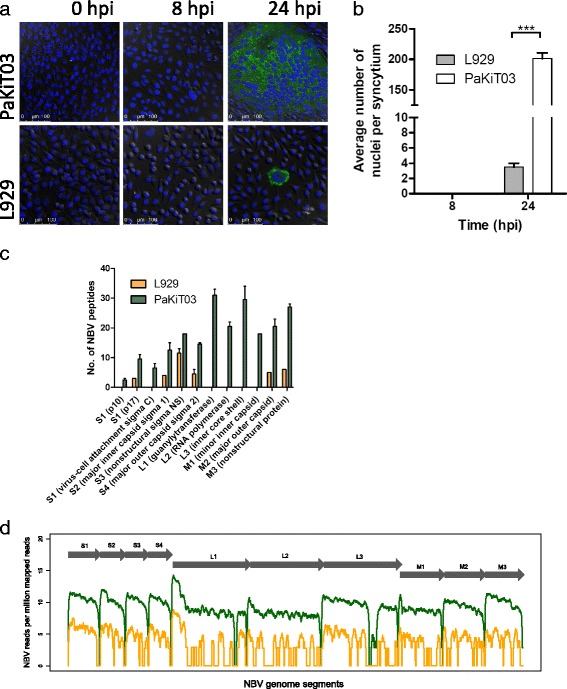
NBV infection of SILAC adapted PaKiT03 and L929 cells at MOI 1.5. **a** Syncytia observed in PaKiT03 cells are progressively larger while significantly smaller syncytia occur in L929 cells. Visualised by immunofluorescence microscopy (DAPI in *blue* and NBV σ proteins in *green*). **b** There is a higher average number of nuclei per syncytium in PaKiT03 cells compared to L929 cells. **c** The number of NBV peptides identified between PaKiT03 and L929 cells at 24 hpi indicates more NBV peptides present in PaKiT03 cells (*green*) compared to L929 cells (*yellow*). **d** The number of NBV mapped reads shows more reads for every NBV segment in the infection of PaKiT03 cells (*green*) compared to L929 cells (*yellow*). ***p < 0.001

The SILAC adapted cells were infected with NBV and infection confirmed by immunofluorescence microscopy. At 24 hpi there was large syncytial events formed in the PaKiT03 cultures encompassing numerous cell nuclei, while in the infected L929 cultures only a few cells were infected and the syncytial events were not as pronounced (Fig. 2a). The infection of SILAC adapted cell types was consistent with previously reported differences in infection kinetics for non-adapted cell types [12]. The number of nuclei per syncytium was quantified in both cell types and was significantly higher in the PaKiT03 cells compared to the L929 cells at 24 hpi (Fig. 2b). The number of NBV peptides (proteome analysis, Fig. 2c) and NBV sequence reads (transcriptome analysis, Fig. 2d) were also significantly higher in the PaKiT03 cells compared to the L929 cells at 24 hpi. At 8 hpi no major syncytial events were observed in either cell line. A small number of single infected cells were observed in PaKiT03 cells at 8 hpi (data not shown). In line with this finding, we also observed very few NBV sequence reads or peptides at 8 hpi in either cell line (data not shown).

### Transcripts and proteins are differentially regulated in response to NBV infection

#### Differentially regulated genes

A summary of the number of RNAseq reads obtained for each sample/replicate is provided as Additional file 3: Table S2. From these paired end reads, 368,115 transcripts for PaKiT03 and 222,549 transcripts for L929 were assembled *de novo*. The *de novo* assembled transcriptomes underwent a BLASTx (≤ e-value 1.0^−5^ threshold) search against the UniprotKB/Swiss-Prot database to identify and annotate transcripts based on sequence homology. To assess transcript differential expression, RNAseq reads from 0, 8 and 24 hpi were mapped back to their respective transcriptomes and differential expression testing was performed using DESeq relative to 0 hpi (control).

**Fig. 3:**
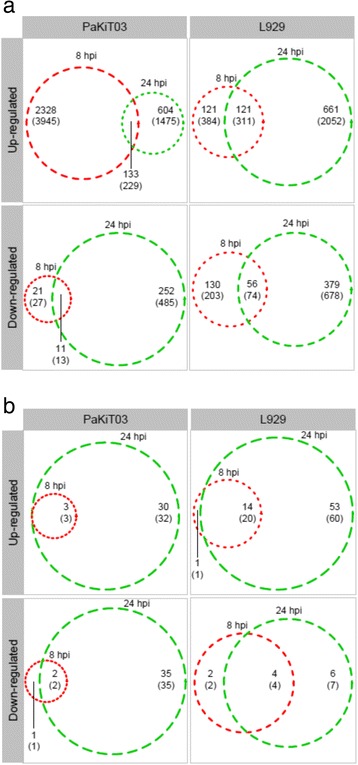
Assessment of differential regulation of transcripts and proteins. **a** Genes and transcripts up- and down-regulated at 8 and 24 hpi with NBV. Transcripts were deemed significantly differentially expressed if their *q*-value (adjusted *p*-value) < 0.05 and had ≥2-fold change (up or down). Values shown in parenthesis represent the number of differentially expressed transcripts (including redundancy), whereas values shown above represent the number of differentially expressed genes (transcript redundancy removed). **b** Proteins were assessed as differentially regulated if they had a ≥ 2-fold change (up or down) relative to 0 hpi. The value in parenthesis indicates the total number of proteins differentially expressed, while the value above signifies the number of non-redundant proteins differentially expressed

The broad transcriptome response varied between the PaKiT03 and L929 cells. PaKiT03 cells demonstrated a robust up-regulation of transcripts at 8 hpi, and then less at 24 hpi. In contrast, L929 cells up-regulated more transcripts at 24 hpi compared to 8 hpi (Fig. 3a). Furthermore, both cell types up-regulated unique sets of transcripts at 8 and 24 hpi, with only moderate overlap between these time-points. Both cell types had more transcripts down-regulated at 24 hpi compared to 8 hpi. As expected significant redundancy was observed in the sets of differentially expressed transcripts. That is, multiple transcripts for many genes were up- or down-regulated. Based on the BLAST annotation for each transcript, we collapsed the list of differentially expressed transcripts into lists of differentially expressed genes (Fig. 3a). Lists and expression statistics for significantly differentially expressed transcripts from PaKiT03 and L929 cells are provided as Additional file 4: Table S3 and Additional file 5: Table S4 respectively.

A subset of differentially expressed genes detected by RNASeq were examined by qPCR to determine the validity of the analysis. The genes investigated were, *Cxcl10*, *Cxcl11*, *Ifit1* and *Ifit3*. In both cell types, the analysis of gene expression by qPCR generally showed expression profile similar to those determined by RNASeq, However, most of the fold changes determined by qPCR were lower compared to those from the RNASeq analysis (Additional file 6: Figure S2).

#### Differentially regulated proteins

Mass spectra obtained for two biological replicates were searched against the translated *de novo* assembled transcriptomes for each cell type. Following strict filtering (see methods), a total of 2072 and 2554 unambiguous proteins were identified in the PaKiT03 and L929 cells, respectively. Only proteins with an average ≥ 2-fold change (up or down) at 8 and/or 24 hpi compared to 0 hpi were deemed significantly differentially expressed. Compared to the number of transcripts/genes differentially expressed, far fewer proteins were differentially expressed in either cell type at 8 and/or 24 hpi. In both cell types, more proteins were up- and down-regulated at 24 hpi compared to 8 hpi. In most cases the proteins up-regulated at 8 hpi were also up-regulated at 24 hpi (Fig. 3b). The proteinGroups files from MaxQuant for PaKiT03 and L929 cells are provided as Additional file 7: Table S5 and Additional file 8: Table S6, respectively.

### Host protein up-regulation is associated with increased transcript expression

**Fig. 4:**
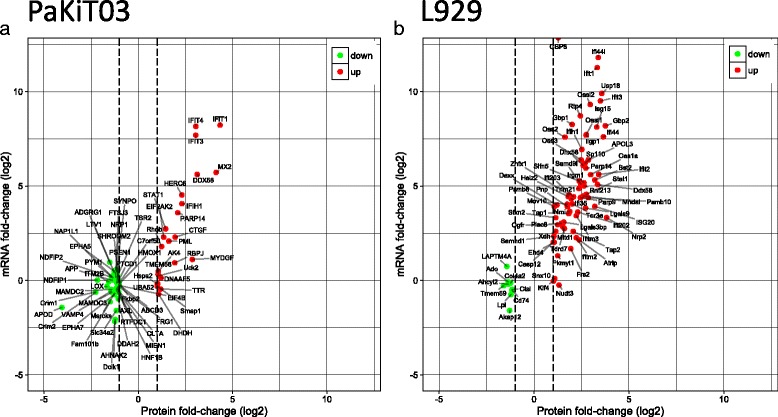
Comparison of transcripts versus proteins fold changes. Comparison of transcript versus protein expression. The relationship between transcript and protein expression was investigated for all proteins up- or down-regulated at 24 hpi in (**a**) PaKiT03 and (**b**) L929 cells. For proteins that had multiple transcripts, the average log_2_ fold-change was calculated for all transcripts belonging to that protein. Both protein and transcript expression is presented as a log_2_ fold-change (relative to 0 hpi). *Vertical dashed* lines represent the 2-fold (1 fold log_2_) threshold for proteins deemed as up- (*red points*) and down-regulated (*green points*)

By virtue of the PIT approach we were able to examine the relationship between gene and protein expression in response to NBV infection. For this analysis, we calculated the average mRNA fold change for all transcripts that were assigned to differentially expressed proteins. The proteins were annotated based on their transcript annotation (determined through BLASTx as described above). Given only a small number of proteins were differentially expressed at 8 hpi, we restricted this analysis to only those proteins that were differentially expressed at 24 hpi. In general, we found a positive relationship between protein up-regulation and mRNA up-regulation in both cell types (Fig. 4a and b). That is, most proteins up-regulated were also up-regulated at the mRNA level. Furthermore, many of the proteins up-regulated in L929 cells at 24 hpi were also up-regulated in the PaKiT03 cells (Fig. 4a and b). In contrast, and in both cell types, proteins down-regulated at 24 hpi were generally not down-regulated at the mRNA level. Taken together these findings suggest that increased protein expression within the host is likely regulated through mRNA transcriptional activity in response to NBV. In contrast, the processes leading to the down-regulation of host proteins following NBV infection are likely driven at the post-transcriptional level.

### Pathway analysis

**Fig. 5:**
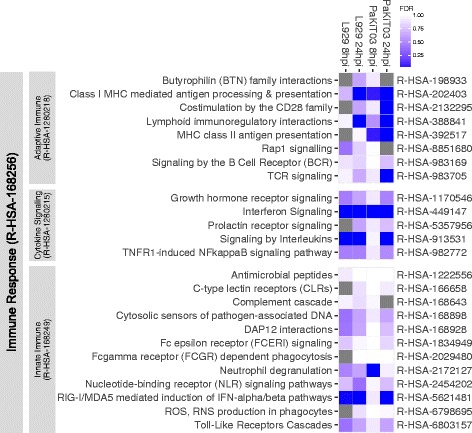
Reactome pathway analysis of up-regulated genes. The most significantly overrepresented immune pathways from L929 and PaKiT03 cells are illustrated with their corresponding FDR values. The broad Immune Response pathway is subdivided into the lower level pathways Cytokine Signaling, Innate Response and Adaptive Response, which are further divided into the sub-pathways shown. *Grey tiles* indicate where no enrichment was observed. The FDR value represents the over-representation probability corrected for false discovery rate and was calculated by Fabregat et al. [34]

To identify the major biological processes differentially regulated in response to NBV infection, we performed pathway analysis using the Reactome database [33, 34]. Given only a moderate number of proteins were up-regulated – and the fact that the majority of those proteins were also up-regulated at the mRNA level – we restricted this analysis to only the gene expression data set. In both the L929 and PaKiT03 cells, overrepresented pathways were identified in the up-regulated gene sets at 8 and 24 hpi. A full description of enriched pathways for L929 and PaKiT03 cells are included as Additional file 9: Table S7 and Additional file 10: Table S8. In both cell types, the broad ‘Immune Response’ (R-HAS-168256) pathway, including its sub-pathways ‘Adaptive Immune Response’, ‘Cytokine Signalling’ and ‘Innate Immune Response’ were the most significantly overrepresented pathway at both 8 and 24 hpi (Fig. 5). Within these sub-pathways, RIG-I/MDA5 mediated induction of interferon, interferon signalling and MHC class I antigen processing/presentation were the most significantly overrepresented pathways in both cell types (Fig. 5).

In contrast to the pathways identified in the up-regulated gene sets, we observed significantly less enrichment for pathways in the down-regulated gene sets. One exception of particular relevance to NBV was the down-regulation of pathways related to cell-cell junction organisation in the PaKiT03, but not L929 cells. Indeed, the claudin and cadherin genes (*CDH6, CLDN4, CLDN3, CLDN19*), which play important roles in cell-cell adherence, were down-regulated in the PaKiT03 cells. This disruption of cell-cell organisation molecules may be a direct consequence of the extensive cell-fusion and syncytia formation induced by NBV.

#### Immune response

**Fig. 6:**
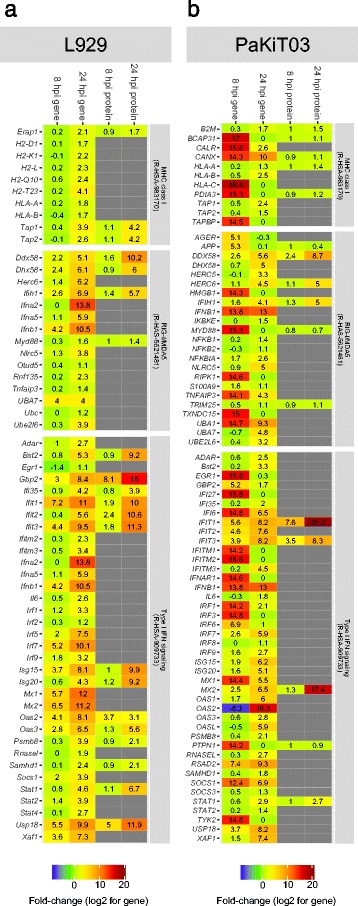
Visualisation of gene and protein expression values. Expression values of (**a**) L929 and (**b**) PaKiT03 genes and proteins at 8 hpi and 24 hpi that are associated with the MHC class I presentation, RIG-I/MDA5 and type I IFN signalling pathways. Fold change is log_2_ for gene expression. Where multiple transcripts were identified only the most significant (lower adjusted *p*-value) expression value was used. *Grey tiles* indicate where no quantitative value was obtained

As described above immune response pathways, and in particular IFN signalling, were significantly overrepresented in both cell types. Subtle differences however existed in the composition of genes and their expression kinetics. Central to the immune response pathways described above is the up-regulation of the intracellular pathogen recognition receptors (namely the RIG-I-like receptors) RIG-I (*DDX58*) and MDA5 (*IFIH1*) and subsequent downstream production of IFN molecules. In both cell types *DDX58/Ddx58* and *IFIH1/Ifih1* were up-regulated at the mRNA and protein level (Fig. 6a and b). Consequently, we also observed an mRNA up-regulation of the transcription factors that are responsible for relaying RIG-I/MDA5 signals to promote the transcription of IFN genes. These genes included IFN regulatory factors (IRFs) and NF-κB genes. In both L929 and PaKiT03 cells, *IFNB1/Ifnb1* was up-regulated significantly at the mRNA level at both 8 and 24 hpi. In L929 cells, we also observed an up-regulation of *Ifna2* and *Ifna5*.

In line with the up-regulation of type I IFN’s, we also observed a significant induction of IFN stimulated genes (ISGs) at both the mRNA and protein level. This included the IFN-induced proteins with tetratricopeptide repeats (*IFIT1/Ifit1, IFIT2/Ifit2* and *IFIT3/Ifit3*), Myxovirus (Influenza virus) resistance genes (*MX1*/*Mx1* and *MX2/Mx2*) and 2-5A synthetase family members (*OAS1, OAS2/Oas2, OAS3/Oas3* and *OASl*) (Fig. 6a and b). Interestingly the kinetics of this response varied between the cell types. Indeed, PaKiT03 cells appeared to show a stronger ISG response at 8 hpi compared to L929 cells for some genes such as the IFN-induced transmembrane proteins (*IFITM1* and *IFITM2/Ifitm2*) and *MX1/Mx1*. The up-regulation of *IFNB1/Ifnb1* was also much stronger within the PaKiT03 cells at 8 hpi compared to L929 cells. We also observed a difference in protein expression between the two cell types. There were 34 and 42 genes in L929 and PaKiT03 cells respectively that were associated with type I IFN signaling. However, there was protein evidence for 14 genes in the L929 cells and 5 genes in the PaKiT03 cells. Genes and proteins related to MHC class I antigen presentation were also significantly up-regulated in both cell types, suggesting activation of cell mediated immune responses.

### Decreasing IFN signalling increases NBV replication in L929 cells

Given the significant up-regulation of type I IFN signalling pathways in L929 and PaKiT03 cells, we then examined the functional effect of IFN signalling on NBV replication and viral mediated fusion. Type I IFN signalling is initiated through the binding of type I IFNs to the type I IFN α/β receptor which is composed of two proteins, IFNAR1/Ifnar1 and IFNAR2/Ifnar2 [36]. We postulated that the strong IFN signalling response in L929 cells may be associated with the reduced viral titre observed in these cells. To investigate this supposition, we first used siRNAs targeted to *Ifnar1* transcript (siIFNAR1) to knockdown the type I IFN signalling pathway. Due to the paucity of bat specific reagents, we could not undertake siRNA experiments within the PaKiT03 cells.

**Fig. 7:**
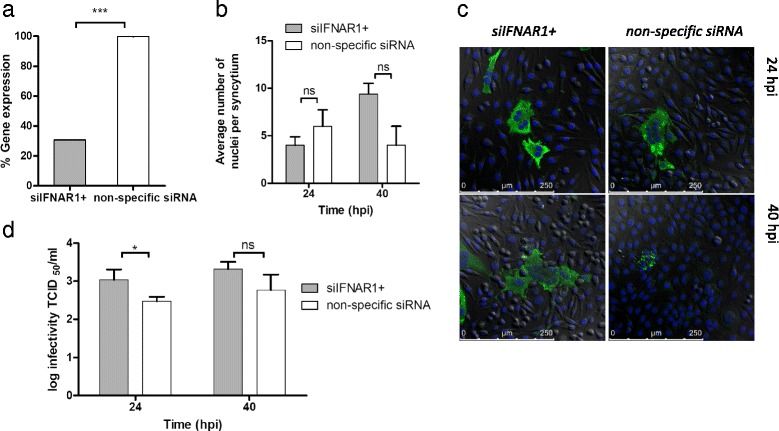
Inhibition of IFN signalling in L929 cells using siRNAs targeted to murine *Ifnar1* (siIFNAR1). **a** Expression of *Ifnar1* following treatment with siIFNAR1. **b** Average number of nuclei per syncytium in siIFNAR treated and non-specific siRNA treated cells infected with NBV at 24 and 40 hpi. **c** Visualisation by immunofluorescence microscopy of siIFNAR1 treated and non-specific siRNA treated cells following infection with NBV at MOI 10 at 24 and 40 hpi. (DAPI in *blue* and NBV σ proteins in *green*). Representative pictures are presented here. *p < 0.05, ***p < 0.001, ns = non-significant. **d** NBV titre following infection at MOI 10 in siIFNAR1+ and non-specific siRNA L929 cells (error bars showing standard error from the mean, *n* = 3)

The efficiency of the knockdown was quantified by qPCR and it was confirmed that after 48 h transfection with siIFNAR1 there was a significant (*p*-value ≤0.001) decrease (approximately 70%) in gene expression of *Ifnar1* in comparison to cells transfected with non-specific siRNA (Fig. 7a). The effects of *Ifnar1* knockdown on viral replication and cytopathic effects was assessed at 24 and 40 hpi. These time points were chosen because they represent the earliest time point when viral induced cell fusion is seen in L929 cells (24 hpi), as well as a later time point when increased viral replication has been seen previously [12].

The average number of nuclei per syncytium was quantified following infection of L929 cells treated with siIFNAR1 and with non-specific siRNA. At 24 hpi in siIFNAR1 treated L929 cells there was a lower but not statistically significant average number of nuclei per syncytium compared to cells treated with the non-specific siRNA. However, by 40 hpi the L929 cells treated with siIFNAR1 had a higher average number of nuclei per syncytium compared to non-specific siRNA treated cells (Fig. 7b).

The progression of syncytia formation during NBV infection was also evaluated by immunofluorescence microscopy through the detection of NBV σ proteins in *Ifnar1* knockdown cells. At 24 hpi, cells infected with virus were fusing with neighbouring cells. By 40 hpi, an increase in the number of nuclei contributing to syncytia were observed in the *Ifnar1* knockdown cells. In contrast, the L929 cells treated with non-specific siRNA showed no increase in the number or syncytia compared to the previous time point, 24 hpi (Fig. 7c). The effect of *Ifnar1* knockdown on efficiency of NBV replication in L929 cells was statistically significant (*p* ≤ 0.05) with an increase in virus titre (half a log) at 24 hpi in siIFNAR1 treated cells but not at 40 hpi (Fig. 7d).

### Increasing IFN signalling delays NBV replication in PaKiT03 cells

As inhibition of IFN signalling resulted in an increased NBV infection, it was hypothesised that enhancing IFN signalling in PaKiT03 cells would reduce NBV replication. Thus, the type I IFN signalling pathway was induced in PaKiT03 cells by treatment with UIFN. Previous studies on *P. alecto* cells have shown that treatment of UIFN for 3 h induces the expression of IFN stimulated genes, ISG54 and ISG56, members of the IFN signalling pathway to at least 5 h post treatment [37].

**Fig. 8:**
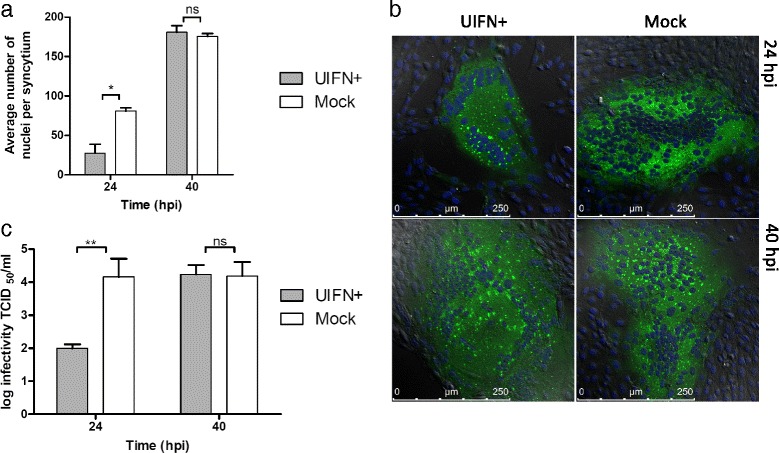
Stimulation of IFN signalling with UIFN in PaKiT03 cells. **a** Quantitation of the number of nuclei per syncytium in UIFN treated and mock PaKiT03 cells. **b** Visualisation by immunofluorescence microscopy of PaKiT03 cells treated with UIFN and mock following NBV infection at 24 and 40 hpi (DAPI in *blue* and NBV σ proteins in *green*). Representative pictures are shown here. **p < 0.01, ***p < 0.001, ns = non-significant **c** NBV titre at 24 and 40 hpi following 3 h simulation with UIFN of PaKiT03 cells prior to virus infection

The progression of virus infection following treatment with UIFN was also assessed by syncytia formation and the average number of nuclei per syncytium was quantified. These observations were confirmed by the intensity of detectable virus σ proteins at 24 hpi under immunofluorescence microscopy following UIFN treatment. At 24 hpi there was a significant increase in the average number of nuclei contributing to syncytia in mock cells compared to treated cells. This was also observed by immunofluorescence microscopy with a decrease in the abundance of virus σ proteins in treated cells. However, by 40 hpi, large syncytia encompassing numerous nuclei were observed in both treated and mock cells and quantitation of the average number of nuclei per syncytium was similar (Fig. 8a and b).

The increased induction of IFN signalling had an effect on virus replication in PaKiT03 cells. At 24 hpi in UIFN treated cells, the virus titre increased to 10^1.99^ TCID_50_/ml but was lower compared to mock cells with a titre of 10^4.16^ TCID_50_/ml. At 40 hpi the virus titre in both treated and mock cells was similar with virus titres of 10^4.24^ TCID_50_/ml and 10^4.19^ TCID_50_/ml, respectively, indicating that the induction of the IFN pathway was declining (Fig. 8c).

## Discussion

Bats are now recognised as a major reservoir of important zoonotic viruses. High profile examples include the henipaviruses, SARS [38] and MERS [39] coronaviruses, and the filoviruses, Ebola [40] and Marburg [41]. Bat borne *Pteropine orthoreoviruses* have also been associated with human disease and represents a growing public health concern. Despite its isolation almost four decades ago, little is known concerning the zoonotic potential of NBV. Our previous work has shown that NBV can infect and replicate in a wide variety of cell lines, and cause extensive fusogenic cytopathic effects in permissive cell lines. The present study, aimed to investigate the host response further by identifying the molecular events that underpin NBV infection and syncytia formation. We proposed that by exploiting two cell types which differ in their susceptibility to NBV, we may also be able to identify host factors that influence cell tropism. Interestingly, despite the fact that the cell lines differed considerable in their ability to support NBV replication, the host response of PaKiT03 and L929 cells was remarkably similar. Immune response processes, namely IFN production and signalling, were highly up-regulated at the mRNA and protein level in both cell types. Indeed, genes and proteins involved in RIG-I/MDA5 pathogen recognition receptors; IFN production; and ISG stimulation were induced at either 8 hpi and/or 24 hpi.

While both cell types showed an overall similar response in respect to immune system process, the expression kinetics – particularly at the mRNA level – varied between the cell lines. Indeed, PaKiT03 cells demonstrated a stronger up-regulation of *IFNB1* and the ISGs (*MX1*, *IFITM2* and *IFITM3*) at 8 hpi compared to the L929 cells at the same time point. We suspect that the early (8 hpi) induction of *IFNB1* and its downstream signalling in the PaKiT03 cells may be a consequence of the increased viral transcription/replication within these cells compared to L929 cells. In line with previously published work [42], we observed no induction of IFN-α in the PaKiT03 cells, while both *Ifna2* and *Ifna5* were induced in the L929 cells at 24 hpi. This is similar to previous work from our laboratory that has shown that the transcript of IFN-α is constitutively expressed in *P. alecto* cells and tissues, but is not inducible following viral infection in PaKiT03 cells [42]. The fact that *IFNB1* was both more rapidly and strongly induced in PaKiT03 cells may suggest that this is a way these cells compensate for a lack of inducible IFN-α. Additional research will be required in the future to explore this hypothesis.

Infection of cells with virus results in a large induction of ISGs. Interestingly, ISG15 that has known antiviral activity against a number of viruses such as Respiratory syncytial virus [43], Chikungunya virus [44], Ebola virus [45] and MRVs [46] was strongly up-regulated in PaKiT03 and L929 cells at the gene level. This expression of ISG15 is evident in the L929 cells at a protein level, however, there was no evidence of protein expression in the PaKiT03 cells. The antiviral activity of ISG15 is through the conjugation to other proteins, altering its function, and is termed ISGylation. Given the effect that ISG15 has on a broad range of viruses, perhaps there is a similar interaction occurring in L929 cells and not in PaKiT03 cells with NBV infection. As there is no evidence of ISG15 expression in PaKiT03 cells which exhibits extensive CPE and high virus titre compared to L929 cells, adds credence to this theory [47].

This is not the first study to show that reoviruses can induce IFN production and signalling pathways. Indeed, mammalian reovirus dsRNA has been shown to be recognised by RIG-I and MDA5 and causes an induction of the IFN response [48]. Not only that, reoviruses are also susceptible to the effects of this IFN response [49].

The proteomic response following infection of HEK293 and HeLa cells with mammalian reovirus serotypes Lang (T1L) and Dearing (T3D) respectively has also been studied [50–52]. Not surprisingly, an induction of proteins associated with the immune response (ISG15/IFIT1/STAT1) was observed. However, the infection of HeLa cells with T1L and T3D resulted in different proteomic profiles, with T3D showing a strong induction of proteins involved in immune regulation compared to T1L. This is seemingly due to the lethal infection caused by T3D compared to T1L [53].

In our study, the IFN response was the dominant biological process up-regulated in both cells types. The differing level of infection/CPE observed in the two cell types did not appear to correlate with the IFN response. That is, PaKiT03 cells demonstrated a strong IFN response while significant viral transcription, translation and replication occurred. In contrast, L929 cells also showed a strong IFN response, however in the absence of significant viral infection. Given these finding, it appeared unclear whether the IFN response in either cell line was effective at reducing NBV replication. To address this question, we investigated the functional relevance of IFN signalling on NBV replication in L929 and PaKiT03 cells. We showed that NBV replication and CPE was reduced in the PaKiT03 cells (at 24 hpi) when cells were primed with Universal type I IFN. Moreover, disruption of the IFN signalling pathway – through siRNA transfections – increased the replication of NBV within the L929 cells. Taken together, these experiments demonstrated that the type I IFN response is indeed capable of reducing NBV replication in both PaKiT03 and L929 cells. However, given the strong IFN response seen in the PaKiT03 cells, it seems unlikely that the IFN activity is solely responsible for inhibiting NBV replication in the L929 cells. Reduced viral receptor expression, could also contribute to the increased resistance of L929 cells to NBV. However, to date, no host molecules have been identified as receptors that are required for NBV infection in any cell type.

The parallel integration of proteomic and transcriptomic datasets provides multidimensional insights of host-pathogen interactions. Certainly, within our study we found that the majority of proteins up-regulated following NBV infection, were also up-regulated at the mRNA level. However, proteins down-regulated were generally not down-regulated at the mRNA level. Taken together these findings suggest that increased protein expression within the host is likely regulated through mRNA transcriptional activity in response to NBV. In line with this theory, we observed the up-regulation of important host transcription factors that specifically promote the expression of immune gene sets. Indeed, in both cell types we observed up-regulation of signal transducer and activator of transcription (STAT1/Stat1 and STAT2/Stat2) and IFN regulatory factors, IRF [54], both of which are essential for IFN signalling pathways. In contrast, the processes leading to the down-regulation of host proteins following NBV infection may be driven at the post-transcriptional level, or possibly a direct consequence of virus mediated degradation. It has been shown that proteins are able to target cellular translation factors, as is the case during feline calicivirus infection, where virus encoded proteases cleave eukaryotic initiation factor, eIF4G, and inhibits host protein synthesis [55]. Additionally, it has been shown that MRV infection, results in the inhibition of host protein synthesis. This inhibition is a stress response that is initiated by the cell through the recognition of dsRNA with dsRNA kinase, PKR, resulting in the phosphorylation of the translation initiation factor, eIF2α [56]. The phosphorylation limits the formation of the ternary complex eIF2/GTP/tRNAi^met^ that binds with the 40S ribosomal subunit to initiate translation. This in turn results in the cytoplasmic aggregation of cellular mRNAs and translation factors termed stress granules [57]. The stress granules hold mRNAs in a translationally inactive state until the removal of the stressor and recovery of the cell, where the mRNA is again translated [58]. Although this response serves to inhibit virus replication, MRV is able to escape the halt in protein expression by disrupting the stress granules [59]. This strategy allows the translation of viral but not cellular mRNA. In addition, the MRV σ3 protein can interact directly with PKR, preventing its activation and subsequent phosphorylation of eIF2α and in this case, both cellular and viral translation proceeds [59–61].

For a fusogenic virus, one of the hallmarks of NBV infection within permissive cell types is virus mediated cell-cell fusion, or syncytia. Syncytia formation from NBV infection is caused by the p10 protein [14]. As a non-structural protein, p10 is produced during infection and transported to the cell surface mediating fusion between neighbouring cells. Within the present study we detected p10 transcripts and peptides in the PaKiT03 cells, but not L929 cells, at 24 hpi. Interestingly, in line with the significant cell-cell fusion observed in the PaKiT03 cells, we observed a down-regulation of genes involved in cell-cell adherence and junctions. In a normal healthy cell, claudin and cadherin proteins, act to maintain tight cell-cell junctions/organisation [62]. The loss of cell-cell junctions and the formation of multi-nucleated syncytia seen in NBV infected cells, may be directly related to the down-regulation of these genes. The importance of cell-cell fusion and syncytia formation in promoting NBV replication remains unclear. In avian reovirus replication it has been shown that syncytia formation is not an essential step but it enhances the virus induced CPE and the release of virus progeny [63]. We have also shown previously with a low cell density infection with NBV, in an environment not favourable for syncytia formation, replication and syncytia still occurred but at a reduced rate [12]. It therefore seems plausible that intervention strategies which aim to inhibit the fusogenic activity of NBV, through directly targeting p10 or restoring cell-cell organisation, could reduce NBV replication.

While we have shown that IFN signalling can influence NBV replication in both cell types, the exact mechanism still remains to be identified. In addition, the contributing effect of the type II and III IFN response cannot be ruled out as they too are known to have antiviral properties. Elucidation of the IFN mechanism in play will be challenging as it is incredibly diverse and involves many different molecules. Another part of the IFN response that may be playing a role in controlling NBV replication are the IFN-induced transmembrane proteins (IFITMs). These proteins are downstream of IFN signalling and are known to block viral replication and syncytia formation by restricting membrane fusion [64]. *IFITM2/Ifitm2* and *IFITM3/Ifitm3* were up-regulated in both PaKiT03 and L929 cells. The effect on syncytia formation of IFITMs has been demonstrated in HIV infection previously. Here, transfected cells expressing IFITM proteins significantly inhibited HIV spread and syncytia formation by antagonising the virus envelope protein [65]. Interference of membrane fusion affects virus entry and has been demonstrated against a range of viruses including Marburg virus, Ebola virus, SARS-CoV and Influenza A virus [66]. The IFITM proteins have also been shown to restrict infectivity and replication of reoviruses, the only known group of non-enveloped viruses to be affected. Although the exact mechanism of action of IFITMs during the replication of reoviruses is unclear, studies on Ifitm3 proposes that it acts on endosomes that are utilised by the virus during either entry or replication [67].

## Conclusions

Utilising the PIT methodology, we investigated and identified pathways that potentially contribute to the difference in susceptibility between L929 and PaKiT03 cell types during NBV infection. The subsequent functional studies on the IFN signalling pathway demonstrates it influences NBV replication. In addition, our transcriptomic and proteomic data also identified a number of downstream mechanisms during infection for further investigation and may be applicable to NBV and other fusogenic orthoreoviruses within this species.

## Additional files

Additional file 1: Table S1. Table 1: Number of 100 bp paired end reads from RNA Sequencing of L929 and PaKiT03 cells and the subsequent number of annotated transcripts with a ≥ 2-fold change in any direction. (XLSX 10 kb)

Additional file 2: Figure S1. Verification of the incorporation of ^13^C_6_ (medium) or ^13^C_6_ and ^15^N_4_ (heavy) isotopes in L929 (a, b and c) and PaKiT03 (d, e and f) cell types. The unlabelled doubly charged peptide ion of SYELPDGQVITIGNER at 895.95 m/z is shown in green. The successive black arrow indicates the expected mass shift of the peptide ion with the incorporation of carbon and nitrogen labelled isotopes. (a and d) unlabelled peptide ion 895.95 m/z shown in green, with no incorporation of carbon and nitrogen labelled isotopes; (b and e) peptide ion 898.96 m/z shown in orange containing ^13^C_6_ with an expected 6 Da mass shift (medium) from the unlabelled peptide ion; (c and f) peptide ion 900.95 m/z shown in blue, containing ^13^C_6_ and ^15^N_4_ with an expected 10 Da mass shift (heavy) from the unlabelled peptide ion. (PDF 412 kb)

Additional file 3: Table S2. Primer sequences used in PaKiT03 and L929 for Real-time PCR (XLSX 9 kb)

Additional file 4: Table S3. Transcripts with an adjusted *p*-value ≤0.05 and significantly differentially expressed in PaKiT03 cells as determined by Deseq in R at 8 hpi and 24 hpi. Genes are annotated according to BLASTx. (XLSX 1715 kb)

Additional file 5: Table S4. Transcripts with an adjusted *p*-value ≤0.05 and significantly differentially expressed in L929 cells as determined by Deseq in R at 8 hpi and 24 hpi. Genes are annotated according to BLASTx. (XLSX 921 kb)

Additional file 6: Figure S2. Comparison of the expression ratios of selected genes: *Cxcl10, Cxcl11, Ifit3* and *Ifit1,* as determined by RNA sequencing and by qPCR in PaKiT03 and L929 cells (error bars are shown for *n* = 2). *p < 0.05, ***p < 0.001. (PDF 140 kb)

Additional file 7: Table S5. MaxQuant proteinGroups files for PaKiT03 with fold changes at 8 hpi (M/L) and 24 hpi (H/L) for the two replicates. Gene names are included along with the peptide contributing to each protein group. (XLSX 335 kb)

Additional file 8: Table S6. MaxQuant proteinGroups files for L929 with fold changes at 8 hpi (M/L) and 24 hpi (H/L) for the two replicates. Gene names are included along with the peptide contributing to each protein group. (XLSX 401 kb)

Additional file 9: Table S7. Pathway analysis with Reactome of up and down-regulated genes of PaKiT03 at 8 hpi and 24 hpi showing the genes associated with each pathway with included FDR value. (CSV 450 kb)

Additional file 10: Table S8. Pathway analysis with Reactome of up and down-regulated genes of L929 at 8 hpi and 24 hpi showing the genes associated with each pathway with included FDR value. (CSV 349 kb)
