# Supplementary figures and images for: Proteomics informed by transcriptomics for characterising differential cellular susceptibility to Nelson Bay orthoreovirus infection
Source: BMC Genomics. 2017 Aug 14;18:615. doi: 10.1186/s12864-017-3994-x (PMC5556373; doi:10.1186/s12864-017-3994-x)

a

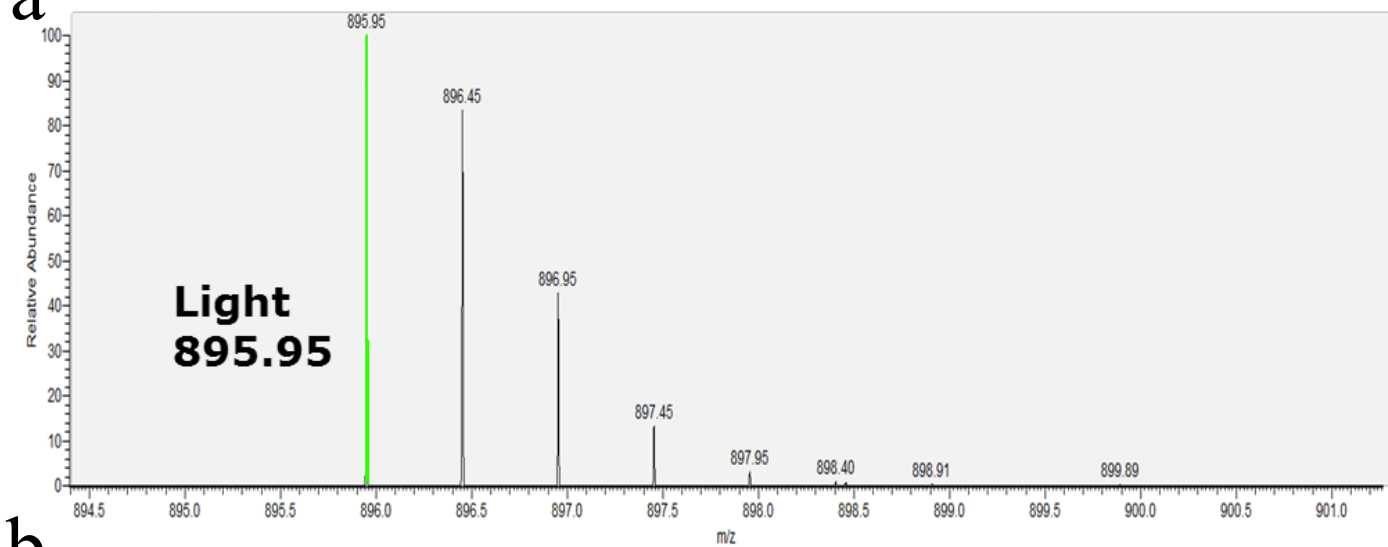

b

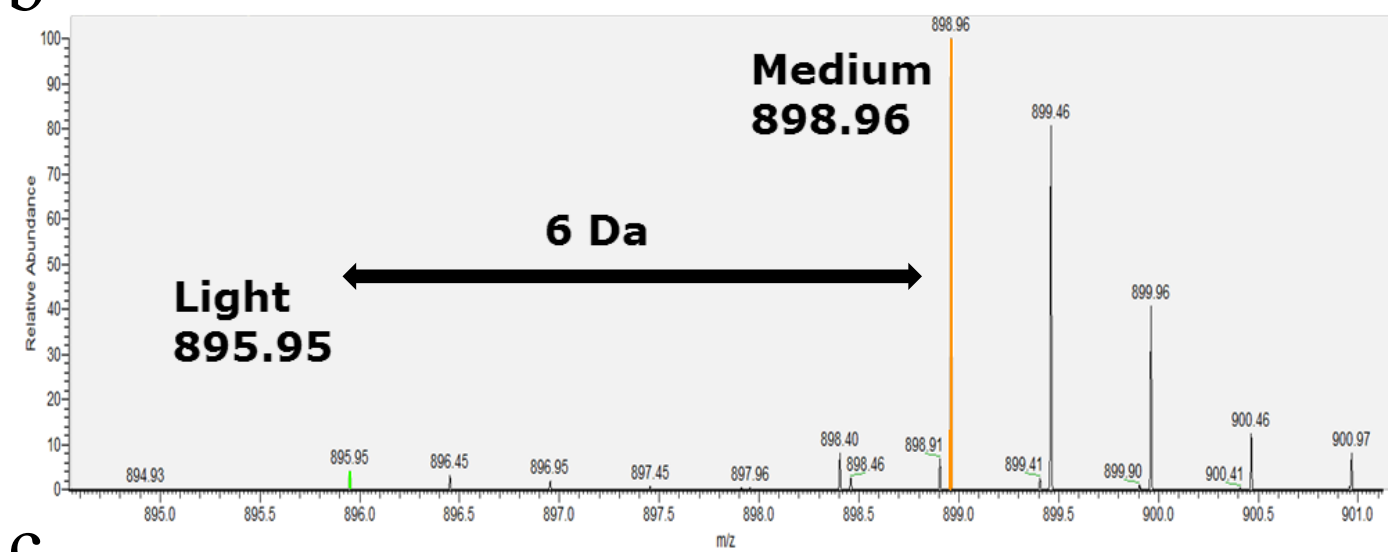

c

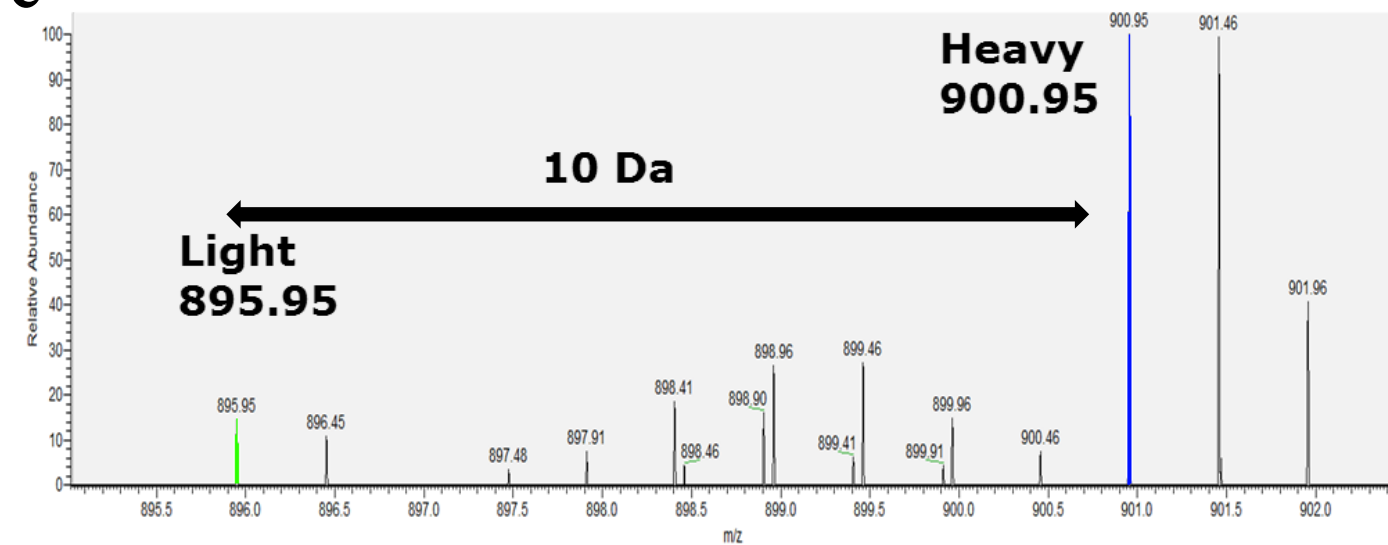

d

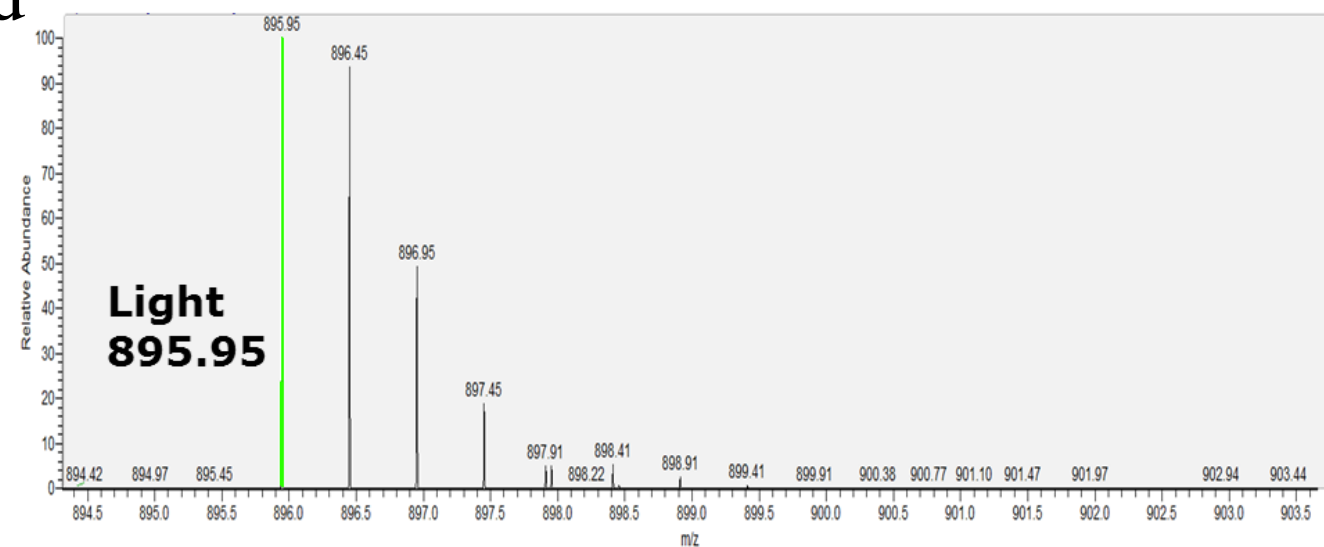

e

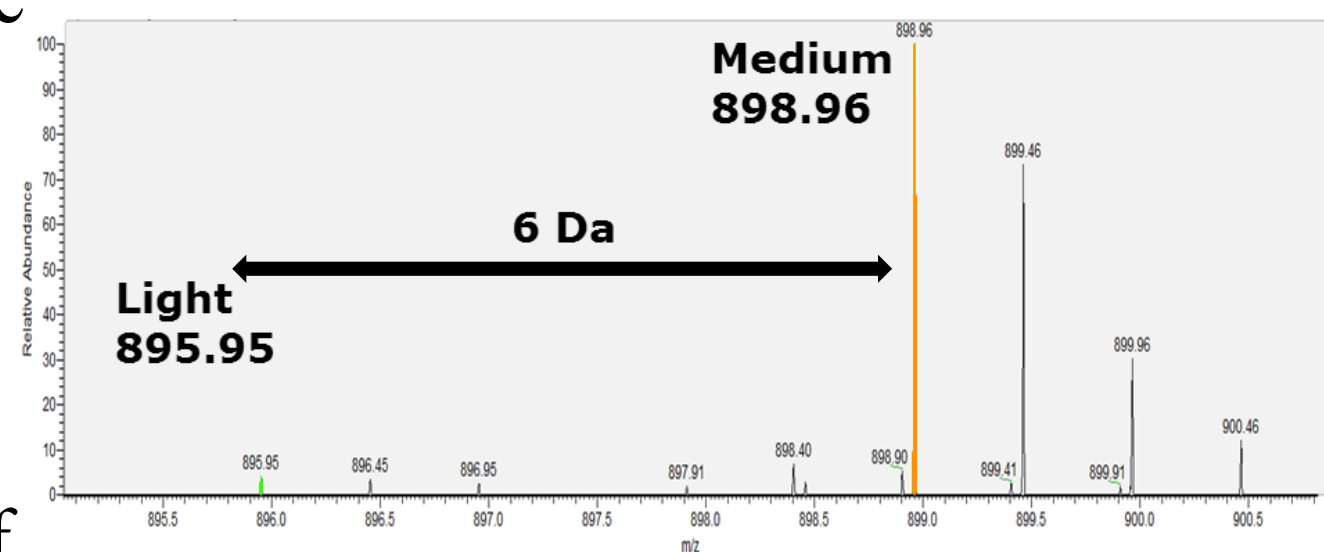

f

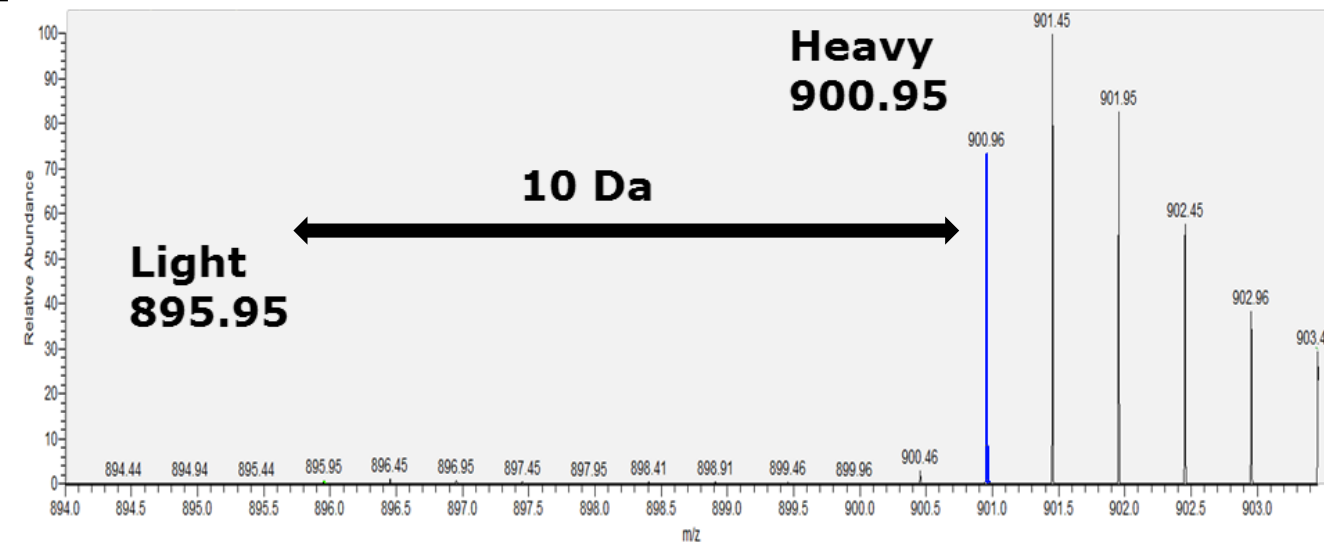

Supplement: Supplementary file 2 — Verification of the incorporation of 13C6 (medium) or 13C6 and 15N4 (heavy) isotopes in L929 (a, b and c) and PaKiT03 (d, e and f) cell types. The unlabelled doubly charged peptide ion of SYELPDGQVITIGNER at 895.95 m/z is shown in green. The successive black arrow indicates the expected mass shift of the peptide ion with the incorporation of carbon and nitrogen labelled isotopes. (a and d) unlabelled peptide ion 895.95 m/z shown in green, with no incorporation of carbon and nitrogen labelled isotopes; (b and e) peptide ion 898.96 m/z shown in orange containing 13C6 with an expected 6 Da mass shift (medium) from the unlabelled peptide ion; (c and f) peptide ion 900.95 m/z shown in blue, containing 13C6 and 15N4 with an expected 10 Da mass shift (heavy) from the unlabelled peptide ion. (PDF 412 kb) [file 12864_2017_3994_MOESM2_ESM.pdf]

## PaKiT03

*Cxcl10*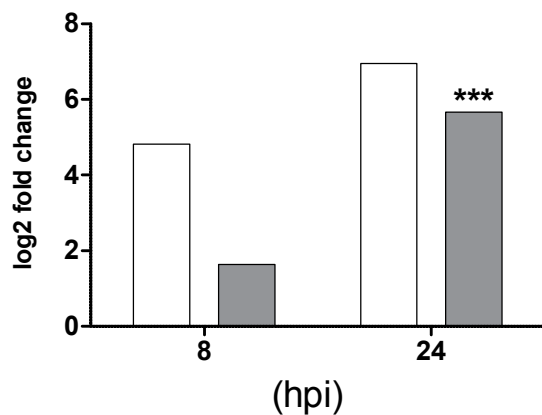

## L929

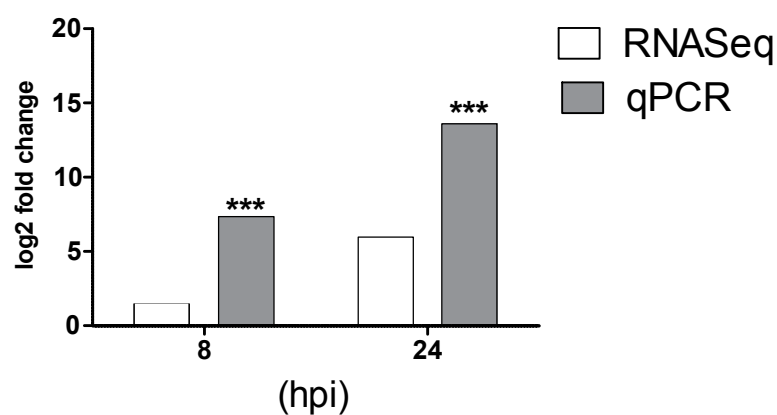*Cxcl11*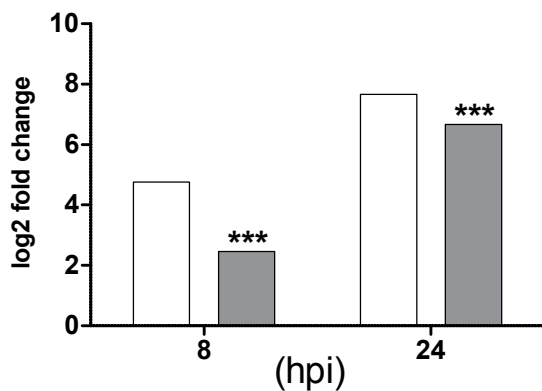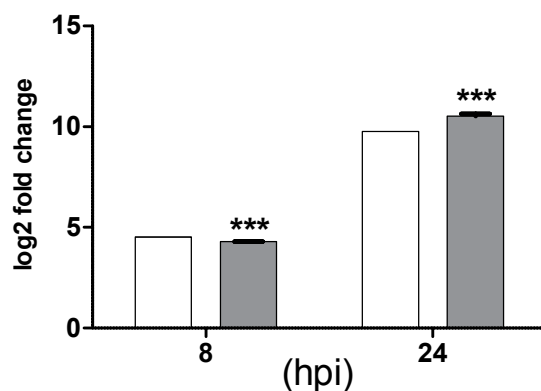*Ifit3*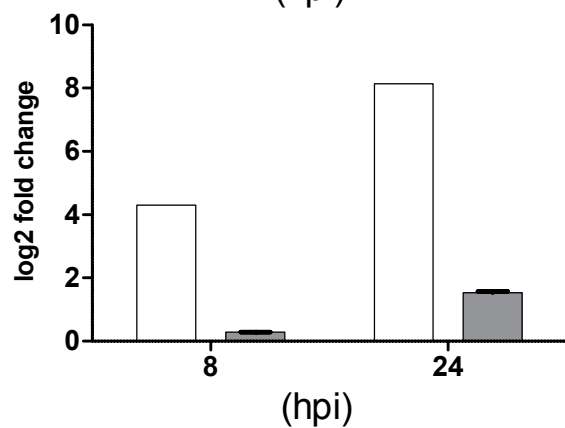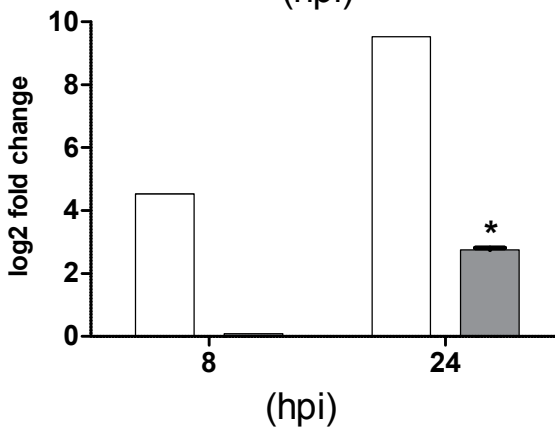*Ifit1*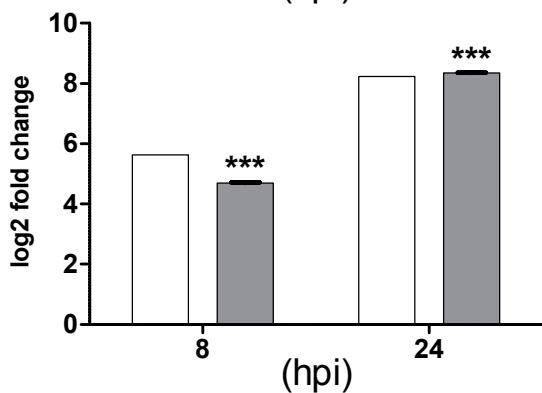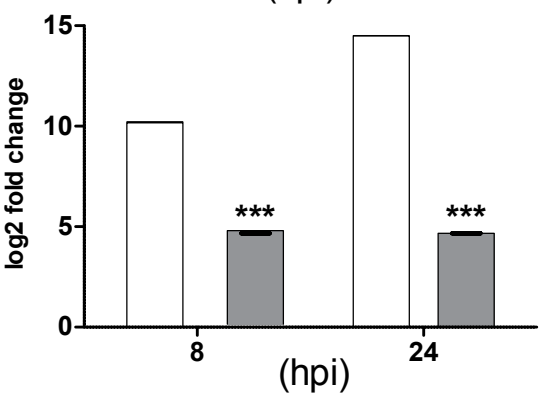

Supplement: Supplementary file 6 — Comparison of the expression ratios of selected genes: Cxcl10, Cxcl11, Ifit3 and Ifit1, as determined by RNA sequencing and by qPCR in PaKiT03 and L929 cells (error bars are shown for n = 2). *p < 0.05, ***p < 0.001. (PDF 140 kb) [file 12864_2017_3994_MOESM6_ESM.pdf]
